# Large‐scale sampling of beetle communities in Laos shows that conversion of natural forests into plantations leads to a decline in family richness and abundance

**DOI:** 10.1002/ece3.10258

**Published:** 2023-07-04

**Authors:** Bounsanong Chouangthavy, Yoan Fourcade

**Affiliations:** ^1^ Entomology Laboratory, Faculty of Agriculture National University of Laos Vientiane Laos; ^2^ Univ. Paris Est Creteil, Sorbonne Université, Univ Paris Cité, CNRS, IRD, INRAE Institut d'écologie et des sciences de l'environnement, IEES Créteil France

**Keywords:** agriculture, beetles, biodiversity, deforestation, landscape contexts, rapid change

## Abstract

Rapid economic development can pose a threat to the biodiversity of tropical countries. In Laos, this is manifested by the conversion of natural forests into plantations, even though this area is one of the biodiversity hotspots of Southeast Asia. Beetle communities can be good indicators of the impact of anthropogenic pressure on natural ecosystems. In this study, we analyzed for the first time a large‐scale inventory of Coleoptera to assess the ecological and anthropogenic drivers of beetle communities in Laos. We examined beetle communities (described at the family level) across the country, located in distinct habitat types, in order to understand the impact of the conversion of natural forest into plantations. We found that beetle abundance had declined in plantations compared to natural forests. At the same time, we observed fewer beetle families in plantations overall, but at the scale of sampling sites there was no difference in local richness compared to natural forests, suggesting a homogenization of beetle communities in anthropogenic habitats. Although results are certainly sensitive to our coarse classification of beetle specimens into families, the negative impact of the conversion of natural tropical forests into agriculture area can still be clearly demonstrated. Our findings highlight that it is possible to make use of unstructured large‐scale inventories to explore how beetle communities responds to landscape changes induced by human activities. We suggest that sampling beetle communities can be used as an ecological indicator to monitor anthropogenic impacts on tropical ecosystems.

## Graphical abstract

The main objective of the present manuscript is therefore to provide information on beetle community composition, diversity, and abundance across a large spatial scale in Laos, focusing on two contrasting landscape contexts.

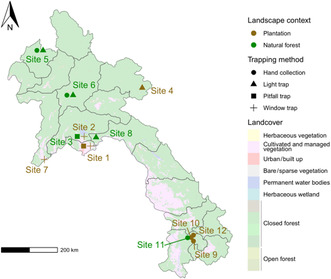

## 1 INTRODUCTION

Southeast Asian tropical forests are home to a rich biodiversity, and are essential habitats and food supply for humans and animals (Estoque et al., 2019). Southeast Asia's tropical lowland rainforests are among the most diverse ecoregions on earth, where a high proportion of endemic species and a high rate of habitat degradation coexist (Myers et al., 2000). However, huge amounts of forests are being destroyed for agricultural cash crops and industrial tree plantations, leading to land use change and intensification that are primarily driven by an increase in the worldwide demand for agricultural products (Kusuma et al., 2018). Insects, for which a global decline has been recently documented (Sánchez‐Bayo & Wyckhuys, 2019), are among the taxa that are known to be affected by deforestation in a tropical context (Correa‐Carmona et al., 2022). Generally, deforestation, agricultural intensification, and climate change, including more frequent extreme weather events, have been suggested as being the major drivers of the global insect decline (Eggleton, 2020; Wagner, 2020). However, this assessment mostly comes from population trends estimated in the Global North (Sánchez‐Bayo & Wyckhuys, 2019), while the current state of insect diversity in tropical contexts remains poorly known.

Laos covers a large part of the Indochinese limestone belt and is one of the most biodiversity‐rich countries of Southeast Asia (Kumar et al., 2016). Its dominant land cover is tropical dense forests, of which approximately 80% are located in mountainous areas with steep to moderate slopes. It includes several National Protect Areas (NPAs) that are considered as biodiversity hotspots, such as the Hin Nam No NPA, which has officially been submitted to become the first natural World Heritage Site of Laos. Many studies report a highly diverse fauna in the region including amphibians, reptiles, birds, bats, and over 100 species of large mammals, new species being frequently discovered (Ceballos & Ehrlich, 2006; MAF & STEA, 2003; MoNRE, 2016; Myers et al., 2000). However, as of yet, no single survey has attempted to describe the whole diversity of Coleoptera, or even insects in general, in Laos. Until fairly recently, the insect fauna of Laos remained one of the most poorly known in Southeast Asia (Sekerka & Geiser, 2016) and existing knowledge mostly comes from specimens collected by foreign visitors before the 1920s. Recently, though, we observed an increase in the number of entomological expeditions, permitted by the country becoming more accessible to foreigners (Chouangthavy et al., 2020).

The extremely rapid economic growth that Laos is experiencing comes at the expense of biodiversity, which is facing a growing number of significant challenges associated with land use changes (World Bank national accounts data, 2017). For example, during the 1990s and 2000s, the land area dedicated to rubber plantations has increased exponentially from 115,732 ha to reach an evaluated surface of 450,000 ha in 2015 (Smith et al., 2020). Such conversion of natural tropical forests into rubber plantations occurs in several tropical regions of the world where it is recognized to negatively impact biodiversity and ecosystems (Warren‐Thomas et al., 2015). Moreover, the economic growth of the region is likely to continue or even accelerate in the near future, as the railway that connects Kunming, China to Bangkok, Thailand, passing through much of Laos, is completed (Chen & Haynes, 2017; Ng et al., 2020). Indeed, infrastructure development will increase the general appeal of the region and encourage foreign investment, contributing to direct and indirect threats to local ecosystems (Borda‐de‐Água et al., 2017; Torres et al., 2016). Knowledge of the influence of human impact, through an effect on landscape structure, on insect diversity in southern Asia, and in Laos in particular, remains insufficient (but see e.g., Chouangthavy et al., 2020).

Beetles (Coleoptera) are the most diverse taxonomic order on Earth. Because they exhibit rich abundance, biomass, and diversity, beetles are often used as indicator species to estimate anthropogenic impact on ecosystems, including in tropical forests (Ghannem et al., 2018; Parikh et al., 2021; Zödl & Wittmann, 2003). For example, dung beetles (Scarabaeidae: Sacarabaeinae) play an important role in the functioning of tropical forest ecosystems while being also sensitive to human disturbance and environmental changes, making them ideal focal species for investigating conservation issues (Slade et al., 2011; Spector, 2006). In southern Asia and in Laos in particular, despite the fact that beetle diversity is high (Moodley et al., 2022) and human activity is growing, the approach of employing beetle community composition, richness, and abundance as surrogates for estimating the impact of agricultural intensification and anthropogenic disturbance has been rarely carried out, especially in Laos. The few studies addressing the question of beetle community richness in relation to human factors were restricted to specific local contexts (Chouangthavy et al., 2020), and were insufficient to estimate more broadly the actual impact of human pressures on beetle biodiversity in Laos. There is thus a need for large‐scale assessments of beetle diversity conducted in natural versus anthropogenic landscapes, in order to estimate the effect of Laos' land use change on its rich biodiversity.

Besides human activity, there is evidence that beetle diversity is also partly structured by climate at large spatial scales (Andrew & Hughes, 2004; Hortal et al., 2011). Even at a more regional scale, beetle assemblages appear to be structured across elevation gradients following the corresponding climatic variation (Dolson et al., 2021; Gebert et al., 2020). This implies that (i) beetle species may be affected by climate change in the recent past and in the future (Harris et al., 2019), and (ii) any attempt to characterize the impact of anthropogenic factors on beetle diversity at a large geographical scale must also account for climatic gradients that may influence the richness, abundance, and composition of beetle communities. Therefore, it is likely that the beetle fauna of Laos, a country that covers a latitudinal gradient of ca. 900 km and hosts four different climate zones (Am, Aw, Cwa, Cwd; Essenwanger, 2001), is somewhat influenced by variation in temperature and precipitation across the country.

In order to understand how the rapid land use change that follows the economic development of the region affects its biodiversity, we investigated beetle community composition, diversity, and abundance across a large spatial scale in Laos (focusing on northern and southern areas, and excluding three provinces in the center), focusing on two contrasting landscape contexts. Specifically, we made use of an unprecedentedly large inventory of beetles carried out in various locations in the country to compare beetle assemblages (characterized at the family level) in natural forests and in plantations, accounting also for climatic gradients that may be an additional driving force of the composition and diversity of beetle communities at macrogeographical scales. We hypothesize that the disturbance caused by anthropogenization results in differences in beetle community composition between natural forests compared to plantations, and that family richness and abundance are higher in natural forests compared to plantations. This work provides an assessment of the effect of landscape context and anthropization on beetle diversity that is still rarely carried out at such a large scale in this region of the world.

## 2 MATERIALS AND METHODS

### 2.1 Landscape context

**FIGURE 1:**
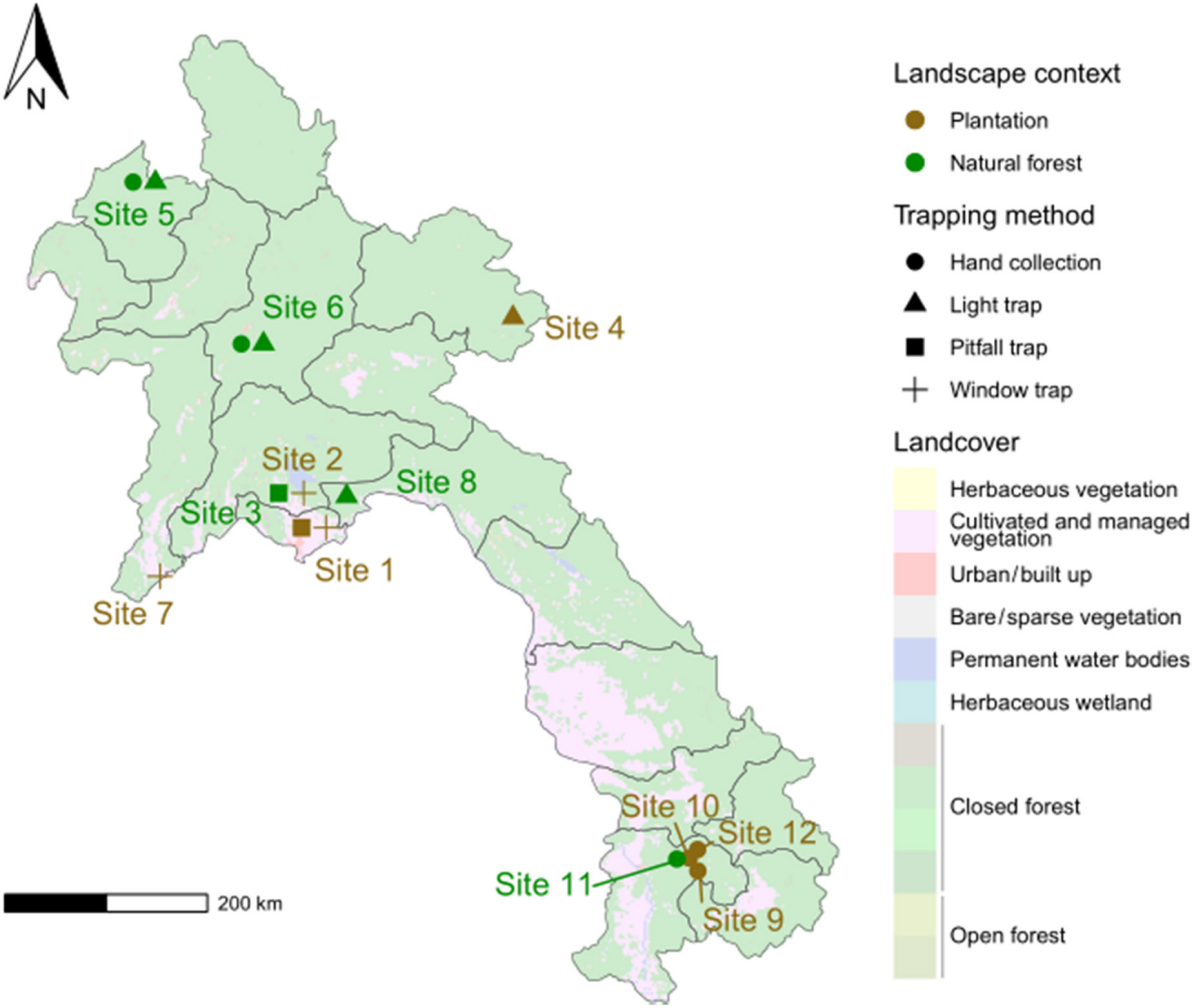
Map showing the distribution of sampling areas in 2018‐19‐20‐21 across Laos, with land cover (from the Copernicus Global Land Service project; data from 2019) shown as background. Note that some sites were sampled using different methods and are thus figured with different shapes.

The study was conducted in 12 locations that cover the northern and southern parts of Laos, leaving only three provinces in central Laos unsampled (Figure 1), in two contrasting landscape contexts (Table 1). Five sampling locations correspond to relatively undisturbed natural forests, which have long been recognized for their outstanding biodiversity. There, the traditional human activity consists mainly of logging, food searching, and hunting. However, natural forests have recently been facing land use intensification pressures, due to extremely rapid economic growth leading to the development of multiple aspects of human activities, including the expansion of agricultural lands. Part of the natural forest in the study areas has been influenced by the construction of a railroad going from the north to the center of Laos, which will be part of a larger railway linking China to Thailand through Laos.

**TABLE 1:** Characteristics of sampling sites, including sampling method and intensity (number of traps and sampling days), environmental variables (mean annual climate and elevation), location, and landscape context (natural forest vs. plantation).

Sites	Trapping methods	Number of traps	Number of sampling days	Mean annual temperature (°C)	Total annual precipitation (mm/m^2^)	Latitude	Longitude	Elevation (m a.s.l.)	Landscape context	Number of sampled individuals	Number of sampled families	Sampling completeness
Site 1	Window	30	60	25.85	1995	18.12	102.79	173	Plantation	4461	30	0.999
Site 1	Pitfall	30	60	25.85	1995	18.12	102.79	173	Plantation	1820	21	0.999
Site 2	Window	30	60	25.54	2344	18.43	102.55	190	Plantation	3926	39	0.999
Site 3	Pitfall	30	60	25.54	2344	18.43	102.55	205	Natural forest	6062	53	1.000
Site 4	Light	1	3	23.15	1831	21.19	101.14	655	Plantation	1023	19	0.999
Site 4	Hand	1	3	23.15	1831	21.19	101.14	655	Plantation	41	4	1.000
Site 5	Light	2	5	22.28	1362	19.99	104.62	368	Natural forest	97	11	0.969
Site 6	Light	1	3	24.52	1454	19.75	102.18	354	Natural forest	142	3	1.000
Site 6	Hand	1	3	24.52	1454	19.75	102.18	354	Natural forest	45	7	0.912
Site 7	Window	15	9	26.05	1347	17.69	101.33	289	Plantation	487	26	0.988
Site 8	Light	1	2	24.32	2552	18.4	103.07	787	Natural forest	784	39	0.991
Site 9	Hand	1	3	20.49	2551	15.24	106.3	1232	Plantation	23	2	1.000
Site 10	Hand	1	3	20.26	2559	15.14	106.28	1313	Plantation	93	2	1.000
Site 11	Hand	1	3	20.35	2548	15.18	106.25	1290	Natural forest	168	4	1.000
Site 12	Hand	1	3	20.35	2548	15.19	106.27	1305	Plantation	48	9	0.939

In contrast, seven sampling locations were located in different types of agricultural landscapes, hereafter referred to as plantations. This type of landscape structure had different agricultural and deforestation histories, and is mostly dedicated to rubber and eucalyptus plantations that have increased exponentially in the country. Part of the present study was undertaken on a large rubber plantation in northern Laos (site 4), which covered 33,000 ha in 2016 and was occupied by various ethnic groups (Kusakabe & Chanthoumphone, 2021); more rubber plantation areas now extend to the center and to the south. Most of the deforestation happened several years before sampling, resulting in a single unfragmented rubber plantation. In addition, sampling also occurred in a eucalyptus plantation planted with a monocrop of eucalyptus trees in rows, the natural forest around plantations being used for extensive livestock and rice paddy field (site 1, 2, and 3). Furthermore, different other types of agricultural activities such as coffee, cabbage, strawberry farms, and grassland mixed with rice paddy fields were covered in the present study across the country (Figure 1).

### 2.2 Beetle sampling and taxonomic assignment

We explored beetle communities across the country in the two distinct habitat types, applying different trapping methods (pitfall traps, window traps, light trapping, and hand collection, in order to capture different aspects of community composition) throughout four consecutive years (2018‐19‐20‐21, depending on sampling site). Pitfall traps consisted of plastic cups (300 cm^3^) with a diameter of 8 cm at the top, buried in the ground so that the top rim was flush with the soil surface. The window traps were made of 1.5‐L transparent plastic drinking water bottles with one window and were suspended upside‐down. The traps were hung on wooden poles at approximately 150 cm above ground level. Both trap types were filled with about 50 mL of 70% alcohol and had a cover for rain protection during the collections carried out in the rainy season. Traps were left 5 days in the field, then all captured insects were brought back to the laboratory, where beetles were separated from other species and debris and sorted into families under a microscope. Light trapping consisted of a 125v bulb and white clothing of 4.5 m^2^ hung up between trees. All beetle specimens were directly stored in 95% alcohol after sorting. Furthermore, hand collection was done by the first author during visits to the field. Because of taxonomic uncertainties and difficulties to identify species precisely, all beetle specimens were assigned to the family level. Note that like several other studies have reported the use of higher insect taxa (i.e., family level) to investigate the impact of habitats and environmental changes (Báldi, 2003; González et al., 2015; Parikh et al., 2021). We then described diversity and community composition across landscape contexts based on the identity and abundance of beetle families in each combination of sampling site, trapping method, and sampling date.

Some sites were sampled each month for several years using different trapping methods and many traps, whereas in some sampling sites collection occurred only occasionally during a few days in a single year, using one or a couple of complementary trapping methods, resulting in a small number of traps. For example, four sampling sites were equipped with pitfall (2 × 30) and window (2 × 30) traps in 2020 (see Table 1). Then, in these sites, sampling occurred each month of the year for 5 days, resulting in a particularly large sampling effort (60 traps × 5 days × 12 months). In contrast, a single sampling site was equipped with 15 window traps for 9 days in 2018. Six sites were sampled by hand collection during a few days in 2019 and 2021. Finally, light trapping was used for 3–5 days in four sampling sites in 2019 and 2021 (Table 1). All beetle specimens were housed at the Entomology Laboratory, Faculty of Agriculture, National University of Laos for future identification.

### 2.3 Climate data

Climate variables used in this study were based on mean annual temperature and precipitation obtained from the Worldclim web portal version 2.1 (Fick & Hijmans, 2017). The variables of Worldclim are raster surfaces derived from the interpolation of weather stations' data collected across the period 1970–2000. We imported a shapefile of Laos boundaries from the GADM database (version 3.6, https://gadm.org/), the coordinates of sampling sites and the rasters of annual precipitation and mean annual temperature from Worldclim into QGIS v.3.22.7. Then, we extracted temperature and precipitation at each sampling locations as a proxy for the climatic variation that exists across sampling sites distributed all over Laos.

### 2.4 Statistical analyses

We first tested the effect of four independent response variables (landscape context [i.e., natural forest vs. plantations], trapping method, temperature, and precipitation) on beetle community composition using a permutational multivariate analysis of variance (PERMANOVA) as implemented in the “vegan” R package (Oksanen et al., 2022), using Jaccard distance as dissimilarity index. We then used non‐metric multidimensional scaling (NMDS) to represent the dissimilarity of beetle communities between natural forest and plantation areas. These analyzes were conducted using species incidence by sampling site and trapping method to reduce the bias due to varying sampling effort.

Subsequently, using now abundance‐based data, we compared family richness between natural forest and plantation areas, by plotting rarefaction and extrapolation curves with the number of collected individuals as a measure of sampling intensity (“iNEXT” package in R v.4.2.1; Hsieh et al., 2016). Additionally, the same procedure was followed to compare family richness obtained by each of the four trapping methods used in our sampling. We also used the asymptotic estimators provided by iNEXT as a measure of the total family richness (including unobserved families) and diversity (Shannon and Simpson indices), in each sampling site, and each trapping method and collection date per site when appropriate. Additionally, we reported sampling completeness by site.

The drivers of the diversity of beetle assemblages were explored with a linear mixed model. The model included the log‐transformed asymptotic estimates of richness obtained in each sampling site, trapping method, and collection date, as a dependent variable. Landscape context, trapping methods, temperature, and precipitation were included as independent fixed variables, while random intercepts included the identity of sampling site nested within the province, and the sampling date. The same approach was used for Shannon and Simpson indices as estimates of the diversity of beetle communities, again using asymptotic estimators from iNEXT. For comparison, we also refitted the same models for an estimator of species richness/Shannon diversity/Simpson diversity that was rarefied/extrapolated for a common sample size equal to the median of observed sample sizes across all sites (*n* = 204).

We used negative binomial generalized linear mixed‐effects model to test the effect of the same variables (landscape context, trapping methods, temperature, and precipitation) on beetle abundances recorded in each sampling site at each date and for each trapping method, using again sampling site identify nested within province and sampling date as random intercepts. Negative binomial models were used because a Poisson generalized linear model that we fitted first showed evidence of overdispersion (dispersion parameter = 0.012, *p*‐value = .032), and the negative binomial model had a lower AIC than the Poisson model (ΔAIC = 2208). Here, we had to account for the very variable sampling effort that produced the observed variation in beetle abundances; therefore, models also included as an offset the number of traps that was used in each sampling site. The same approach was applied first for the total beetle abundance, then for the most abundant beetle families separately: Carabidae, Scarabaeidae, Nitidulidae, Curculionidae, and Chrysomelidae. All statistical analyses were performed in the R platform (version 4.2.1, R Development Core Team, 2022), mixed models being fitted using the “lme4” package (Bates et al., 2015). Overdispersion and model residuals were checked using the “DHARMa” R package (Hartig, 2022). The significance of each term was estimated using Wald Chi‐square tests.

## 3 RESULTS

**FIGURE 2:**
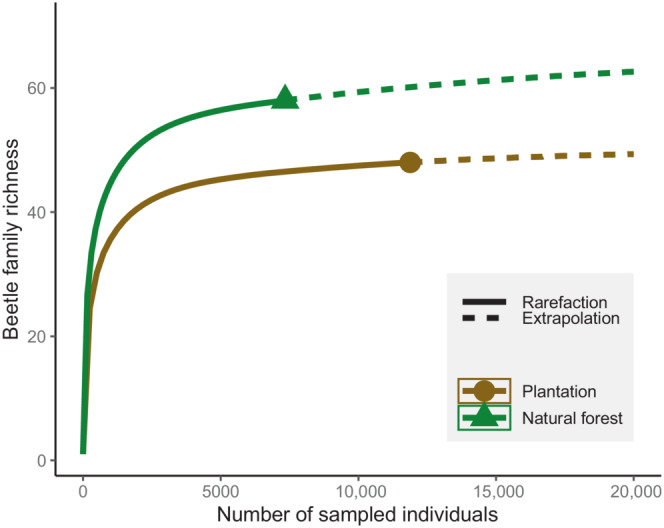
Rarefaction and extrapolation curves of beetle family richness for natural forests and plantation areas.

A total of 19,220 beetle individuals were recorded across all sampling sites during the study period, which represents 63 beetle families, including 58 recorded in natural forests and 48 in plantations. All sites had a sampling completeness >0.91 (Table 1). The rarefaction and extrapolation curves, adjusting for the number of specimens collected in natural forests (7339) and plantation areas (11,881), clearly confirmed that beetle community composition in natural forests had a higher overall cumulative diversity of families compared to plantations (Figure 2). As expected, sampling conducted using different trapping methods did not provide the same number of beetle families, the lowest being achieved by hand collection (Figure S1).

**FIGURE 3:**
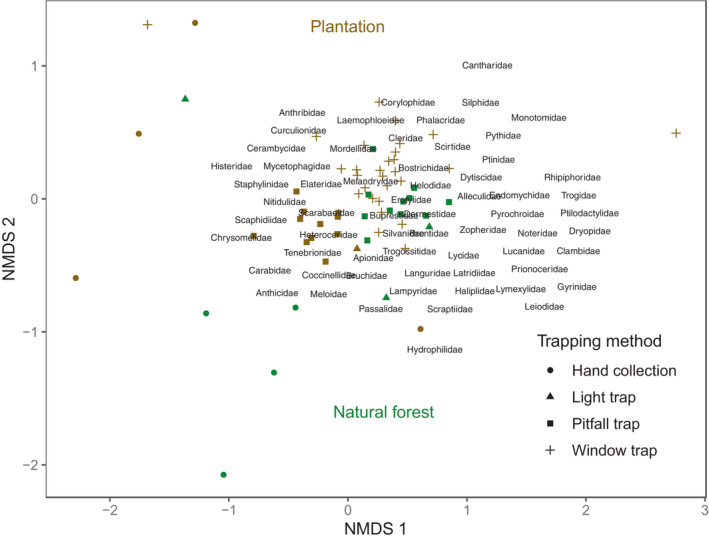
Non‐metric multidimensional scaling (NMDS) representing the pairwise dissimilarity of beetle communities (computed as the Bray‐Curtis distance). Colored dots show the position of each combination of sampling site, date of collection and trapping method in the NMDS ordination, grouped into natural forest and plantation areas.

Beetle community composition appeared to be dependent on the trapping method (*F*_3,56_ = 2.571, *p*‐value < .001), landscape context (*F*_1,56_ = 0.821, *p*‐value < .001), and precipitation (*F*_1_ = 0.426, *p*‐value = .026), but not on temperature (*F*_1,56_ = 0.369, *p*‐value = .0824), according to the PERMANOVA analysis. We noted that the temperature variable becomes significant when this term is included before trapping methods in the PERMANOVA, which may reflect an effect of temperature on community composition. Similarly, visual examination of NMDS confirmed that the beetle families collected using different trapping methods, or collected either in natural forests or in plantation areas, differed but were also largely overlapping (Figure 3).

The richness of beetle communities (in terms of the estimated number of families per site, per date and per trapping method) was found to be dependent on the trapping method (Chi‐square = 8.947, df = 3, *p*‐value = .030; Figure S2). Specifically, the highest richness was sampled in pitfall traps (marginal mean family richness = 31.8; CI 95% = [1.473, 687.6]), while window traps provided the lowest beetle family richness (marginal mean family richness = 10.0; CI 95% = [0.404, 248.9]). It was not, however, influenced by the other three variables of interest (landscape context (Chi‐square = 0.098, df = 1, *p*‐value = .754), temperature (Chi‐square = 0.293, df = 1, *p*‐value = .588), precipitation (Chi‐square = 0.218, df = 1, *p*‐value = .640)). In addition, there was also no significant effect of any variable on beetle community diversity as estimated by Shannon index, but it appeared that Simpson diversity was slightly higher in natural forests compared to plantations (Chi‐square = 4.091, df = 1, *p*‐value = .043; marginal mean = 3.95; CI 95% = [1.890, 8.230] vs. 2.48; CI 95% = [1.160, 5.310], respectively; Table S1 and Figure S2). There was no significant effect of any of the variables tested when richness and diversity indices were estimated for a common sample size.

**FIGURE 4:**
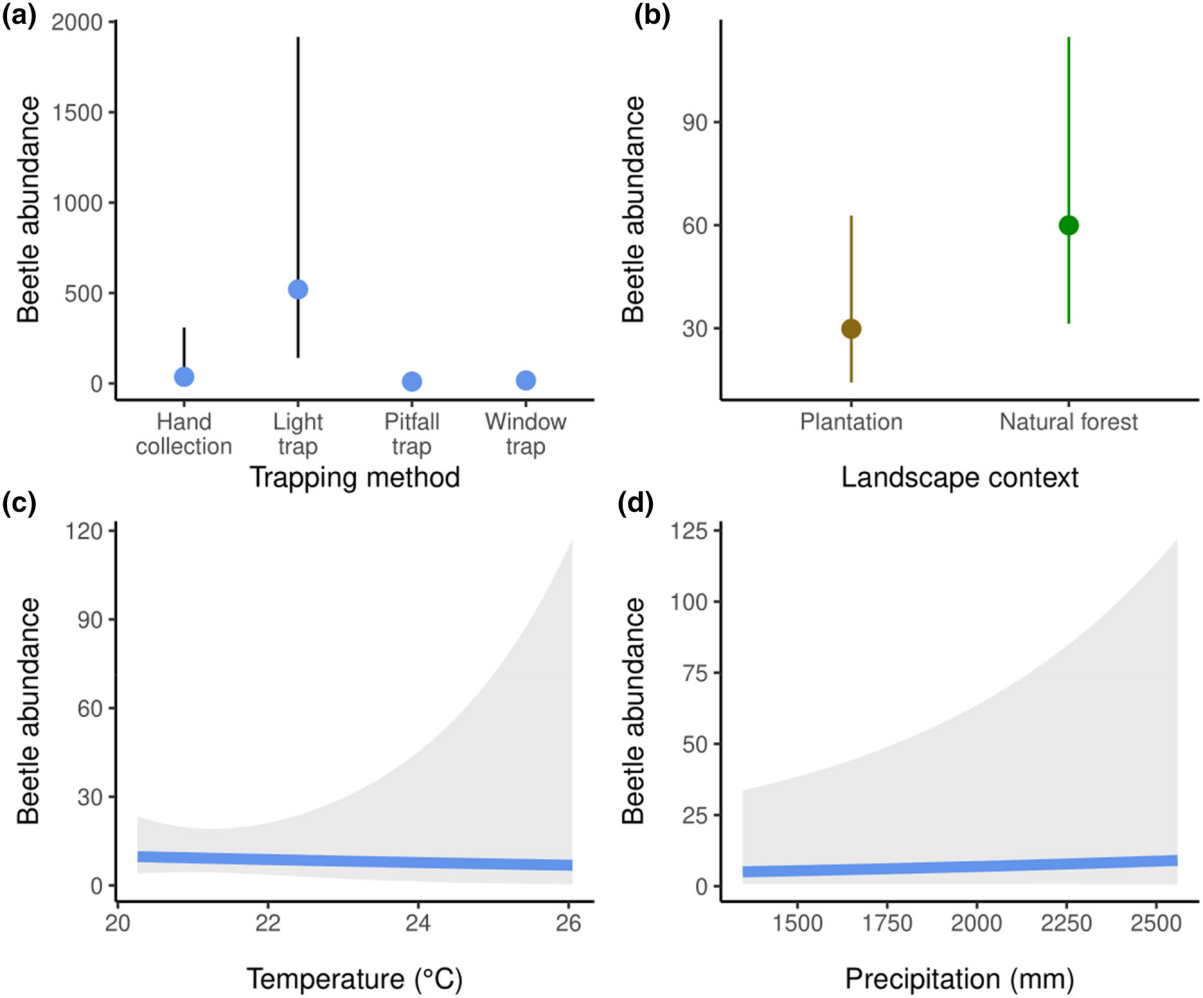
Partial dependence plots showing the predicted beetle abundance for different levels of the four response variables tested (only trapping method and landscape context are significant at the *α* = .05 level), modeled using a negative binomial mixed‐effect model.

There was a strongly significant effect of the trapping method (Chi‐square = 40.319, df = 3, *p*‐value < .001) on the variation of beetle abundance across sites, with light traps being predicted to provide higher abundance (Figure 4). Moreover, the abundance of beetle communities was significantly higher (Chi‐square = 3.892, df = 1, *p*‐value = .049) in natural forests (estimated marginal mean abundance = 60.0; CI 95% = [31.3, 114.7]) compared to plantations (estimated marginal mean abundance = 29.8; CI 95% = [14.2, 62.7]). It was not, however, associated with temperature (Chi‐square = 0.047, df = 1, *p*‐value = 0.829) or precipitation (Chi‐square = 0.860, df = 1, *p*‐value = .354) (Figure 4). When analyzes were computed for five families separately, we similarly identified a significant effect of trapping methods. We were not, however, able to distinguish the effect of landscape context on beetle abundance, except for Scarabaeidae (Table S2) where the predicted abundance were actually higher in plantations (estimated marginal mean abundance = 6.735; CI 95% = [1.959, 23.155]) than in natural forests (estimated marginal mean abundance = 1.007; CI 95% = [0.555, 5.410]).

## 4 DISCUSSION

Despite the fact that tropical areas host the majority of insect diversity, they are generally vastly undersampled compared to temperate ecosystems (Hellmann & Sanders, 2007). This knowledge gap is particularly undesirable in Southeastern Asia, which is experiencing a rapid land use change that may imperil its rich insect biodiversity. In this study, we report for the first time a large sampling of the whole order Coleoptera carried out across a large part of Laos, which enabled the investigation of the drivers of beetle diversity at a large spatial and taxonomical scale. This is a step toward a better understanding of insect diversity in Asian tropical forests, and of the threats they may face.

The present study first confirmed our expectation that beetle diversity is high in the country. From the ca. 20,000 collected specimens, we recorded 63 beetle families. Moreover, more than 50 beetle specimens remained impossible to assign to any known family and were thus excluded from this study. Some of them may belong to additional beetle families, suggesting that the diversity of beetle families in the region may be even larger. It is however hard to reliably estimate the actual number of families living across the various land cover types of the country without a meticulous approach involving standardized sampling protocols and molecular taxonomy (García‐Robledo et al., 2020). The data presented here reveals slightly higher number of families than that of earlier recorded data in surrounding countries such as Thailand, Vietnam, and Hong Kong (Rattanawannee et al., 2013; Thinh et al., 2004; Zhao et al., 2022). Overall, 177 Coleoptera families were recorded globally, meaning that we recorded 36% of all known beetle families (Moodley et al., 2022). However, due to practical constraints, the present study described beetle communities at the family level only. Indeed, beetle taxonomy is notoriously difficult in the absence of detailed identification keys or molecular tools (see e.g., Jin et al., 2020; Sabatelli et al., 2021). The actual diversity at the species level, that is species richness, is thus much more important, and may include endemic or undescribed species.

According to our results, beetle abundance was reduced in plantations compared to natural forests, which is in line with global patterns which show that insects are sensitive to habitat disturbance from human activities such as agricultural expansions or settlements (Hansen et al., 2012; New et al., 2021; Sánchez‐Bayo & Wyckhuys, 2019). Natural forest ecosystems play an important role in species diversity worldwide (Gibson et al., 2011), whereas intensification of agriculture is identified as a major cause of insect diversity decline and extinction (Sánchez‐Bayo & Wyckhuys, 2019), but also soil carbon loss (Guo et al., 2022). A strong impact of land use intensification has been reported for beetles and other insects in tropical forests in Asia, as well as in Africa and America (Phillips et al., 2017). Several observational and experimental studies have revealed that the conversion of natural forests into plantations is harmful to species that cannot adapt to their new environmental conditions (Uribe et al., 2021; Warren‐Thomas et al., 2015); our results show that Laos is no exception. Therefore, a large number of insect communities may be, currently or in the near future, at risk from land use intensification in Laos, even though the region is still mainly covered by mountains and forests.

A more in‐depth understanding of the response of beetle diversity to current and future economic development in Laos is needed to implement practical conservation actions. In this regard, this study clearly demonstrates that the rapid and continuous land use changes in the country is experiencing may threaten beetle communities; not only their abundance declined in plantations, but across the whole survey we also sampled fewer families in plantations compared to natural forests (48 vs. 58 observed families, respectively). This finding is in line with other studies showing the impact human‐modified landscapes can have on beetle biodiversity; for example, dung beetle communities are well recognized as a good indicator to estimate the influence of anthropogenic habitats in tropical forests (Gardner et al., 2008; Halffter & Arellano, 2002). In the present study, we investigated beetle communities across a long latitudinal gradient, in which the northern to center parts are facing a modernization of the road network in addition to the conversion of forests into plantations. In this regard, a recent report by Danyo et al. (2018) pointed out the potential risks of forest and biodiversity loss resulting from road improvement in Laos. We believe that our findings will therefore be useful and important in order to predict the conservation issues arising from land use changes in the region.

The observed difference in richness and abundance between plantations and natural forests were associated with differences in terms of family composition, although it was difficult to detect families that were strongly associated with a type of landscape. Similarly, although fewer families were sampled in plantations at the scale of the whole country, anthropization seems to have relatively little impact on community diversity when analyzed at the scale of each sampling site. Indeed, we did not detect difference in family richness, but only on Simpson diversity index (which confirms the effect on abundance). Logically, this must be caused by a higher homogeneity of communities located in plantations, that is, the same set of families are found in all plantations, while forest communities are more diverse from each other (despite a similar local diversity). Biotic homogenization, in which a few common species takeover specialist species, is frequently observed in human‐modified landscapes (McKinney & Lockwood, 1999), and has also been observed in beetles (Ramírez‐Ponce et al., 2019). However, results may vary at a lower taxonomic level; it is likely that species diversity is actually reduced along human activities, in accordance with previous studies (e.g., Jung & Lee, 2016; Vanbergen et al., 2005). Here, the beetle collection contained a huge number of specimens, and the taxonomy of many groups is challenging due to their complex diagnostic morphological characters and their small body size. An improved dataset that would distinguish individuals at the species‐level may reveal a slightly picture, including perhaps an effect of agricultural development on species diversity at the local scale.

Due to the geographical scale of the study, we found an effect of climate, specifically precipitation, on community composition. It was not, however, reflected by differences in terms of family richness or abundance along the climate gradient. However, again, it could be because the present study has investigated families only and not species. Generally, assessments of climatic niches are considerably more precise when carried out at lower taxonomic levels (Bayliss et al., 2022; Gonzalez et al., 2011), meaning that each individual species may have vastly different responses to climatic variables that would be masked when merged into whole families.

It was clear that both composition and abundance differed depending on the trapping method. Here, we showed that, when all other variables are taken into account, pitfall traps were able to capture a much larger number of beetle families compared to all other methods. However, while light traps were way behind in terms of diversity, they provided a larger sample (in terms of abundance). In this case, a good sampling strategy should probably use several complementary approaches to sample the whole diversity of beetles. For instance, while light traps are probably suitable for sampling flying insects, pitfall traps are adapted for ground‐dwelling species.

## 5 CONCLUSIONS

The present study provides the first approach that attempts at investigating the effect of various independent variables on beetle community composition and richness along contrasted landscape types across Laos. A potential limitation of the study is that sampling did not follow a standardized protocol across the country. On the contrary, we compiled here a dataset of beetle specimens collected using different sampling efforts and methods. We employed various approaches to account for these unequal sampling strategies: country scale and local family richness were estimated from accumulation curves, trapping methods were always included as covariables, and sampling effort was incorporated as an offset in statistical models. The main outcome of our results was that the conversion of natural forests to plantations appeared to be harmful for beetle communities, since less families were found in plantations compared to natural forests overall, and their abundance was reduced locally.

We may gain additional knowledge by comparing beetle communities in pairs of sites that were sampled in similar conditions. Here, site 9 (rubber plantation) and site 11 (natural forest) were located in the same province of Champasak, and were sampled in the same year and using the same method of hand collection. Results of the sampling confirmed our general conclusions, as we found a reduced number of families (2 vs. 4) and abundance (23 vs. 168) in the plantation area compared to the nearby natural forest. More studies are needed to better understand this pattern. This may be achieved in the future by implementing long‐term monitoring of beetles across Laos following a simple protocol that can be used by many volunteers, and by incorporating a better taxonomic resolution (i.e., species) in analyzes. The central part of the country, which we did not sample in this study, would also benefit from a proper inventory of beetles. In this regard, our study shed light on the potential usefulness and difficulties of sampling beetle diversity in southern Asia and in Laos in particular. It is widely accepted that Laos is a hotspot for the biodiversity of beetles, insects, and other organisms. By providing a first large‐scale view of beetle family diversity of Laos, we aim to provide a basis for future studies investigating the impact of an extremely rapid economic change, associated with land use change, on insect diversity in Laos.

## AUTHOR CONTRIBUTIONS

**Bounsanong Chouangthavy:** Conceptualization (equal); data curation (equal); formal analysis (equal); methodology (equal); visualization (equal); writing – original draft (equal); writing – review and editing (equal). **Yoan Fourcade:** Conceptualization (equal); data curation; formal analysis (equal); methodology; software (equal); visualization (equal); writing – review and editing (equal).

## CONFLICT OF INTEREST STATEMENT

The authors do not have any conflicts of interest.

## Supporting information


Data S1

## Data Availability

The beetle sampling data is available in the Figshare repository: https://doi.org/10.6084/m9.figshare.23428457.v1.
