# Supplementary material for: Large‐scale sampling of beetle communities in Laos shows that conversion of natural forests into plantations leads to a decline in family richness and abundance
Source: Ecol Evol. 2023 Jul 4;13(7):e10258. doi: 10.1002/ece3.10258 (PMC10318580; doi:10.1002/ece3.10258)
Supplement: Supplementary file 1 — Data S1 [file ECE3-13-e10258-s001.docx]

**Supporting Information**

**TABLE S1** Effect of trapping method, landscape context (natural forest vs. plantation), temperature and precipitation on Shannon or Simpson diversity indices of beetle communities.

| **Variables** | **LR Chi²** | **df** | **p-value** |
| --- | --- | --- | --- |
| *Shannon* |  |  |  |
| Trapping method | 1.2619 | 3 | 0.7382 |
| Landscape context | 0.3129 | 1 | 0.5759 |
| Temperature | 0.1302 | 1 | 0.7182 |
| Precipitation | 0.0594 | 1 | 0.8075 |
| *Simpson* |  |  |  |
| Trapping method | 1.5638 | 3 | 0.6676 |
| Landscape context | 4.0912 | 1 | 0.0431 |
| Temperature | 0.0014 | 1 | 0.9699 |
| Precipitation | 0.3368 | 1 | 0.5617 |

**TABLE S2** Effect of trapping method, landscape context (natural forest vs. plantation), temperature and precipitation on the abundance of five beetle families, which included Carabidae, Scarabaeidae, Nitidulidae, Curculionidae, and Chrysomelidae.

| **Family** | **Variables** | **LR Chi²** | **df** | **p-value** |
| --- | --- | --- | --- | --- |
| Carabidae | Trapping methods | 42.3270 | 3 | <0.0001 |
|  | Landscape context | 1.2381 | 1 | 0.2658 |
|  | Temperature | 2.4874 | 1 | 0.1148 |
|  | Precipitation | 0.0001 | 1 | 0.9907 |
| Scarabaeidae | Trapping methods | 30.0213 | 3 | <0.0001 |
|  | Landscape context | 5.7986 | 1 | 0.0160 |
|  | Temperature | 0.3972 | 1 | 0.5286 |
|  | Precipitation | 0.2440 | 1 | 0.6213 |
| Nitidulidae | Trapping methods | 47.4962 | 3 | <0.0001 |
|  | Landscape context | 1.2096 | 1 | 0.2714 |
|  | Temperature | 0.1611 | 1 | 0.6882 |
|  | Precipitation | 0.0775 | 1 | 0.7806 |
| Curculionidae | Trapping methods | 27.7715 | 3 | <0.0001 |
|  | Landscape context | 0.7580 | 1 | 0.3839 |
|  | Temperature | 3.5495 | 1 | 0.0596 |
|  | Precipitation | 4.0963 | 1 | 0.0430 |
| Chrysomelidae | Trapping methods | 43.8536 | 3 | <0.0001 |
|  | Landscape context | 0.8676 | 1 | 0.3516 |
|  | Temperature | 3.0045 | 1 | 0.0830 |
|  | Precipitation | 0.0961 | 1 | 0.7566 |


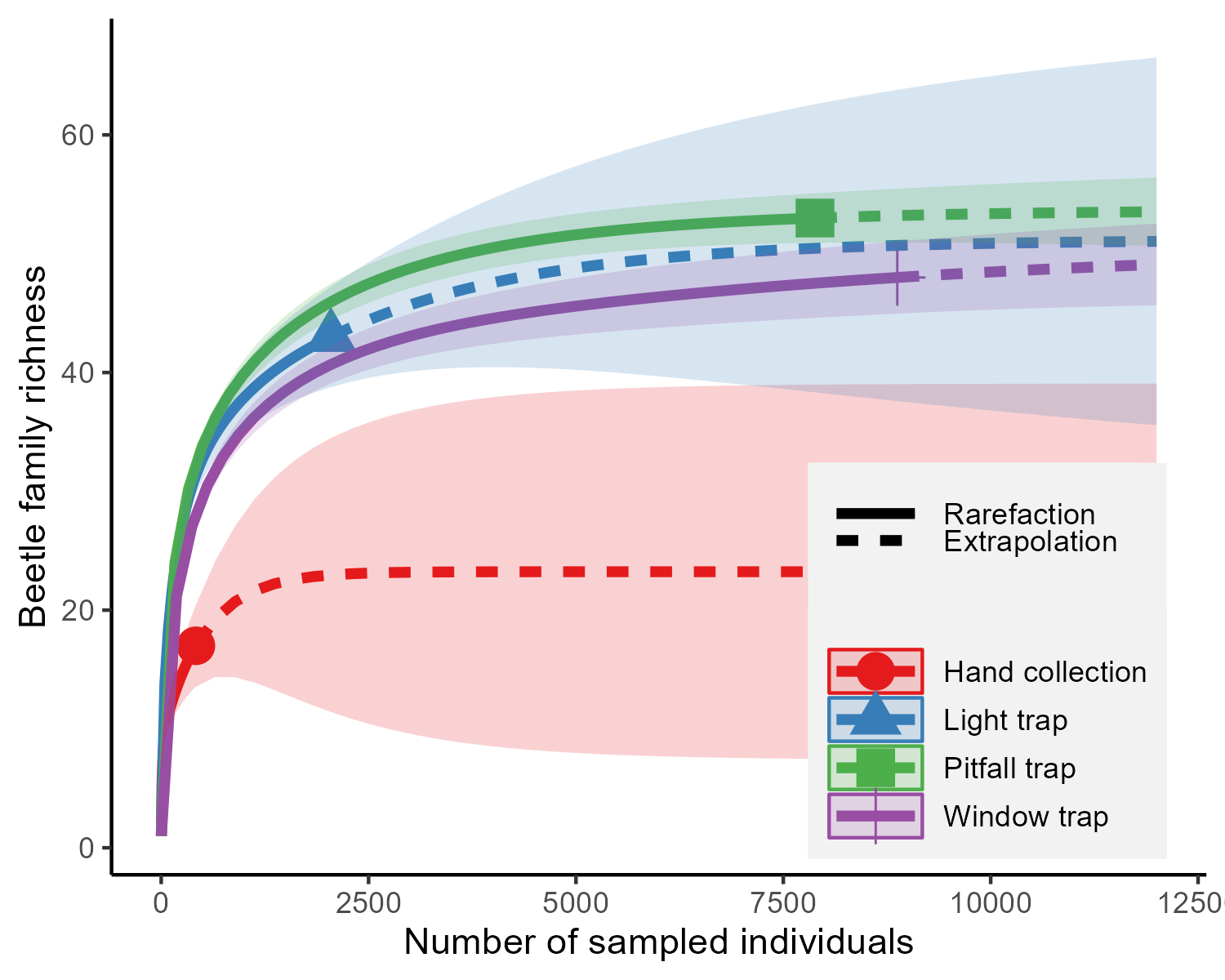


**FIGURE S1:** Rarefaction and extrapolation curves of beetle family richness for each of the four trapping methods used in our sampling.


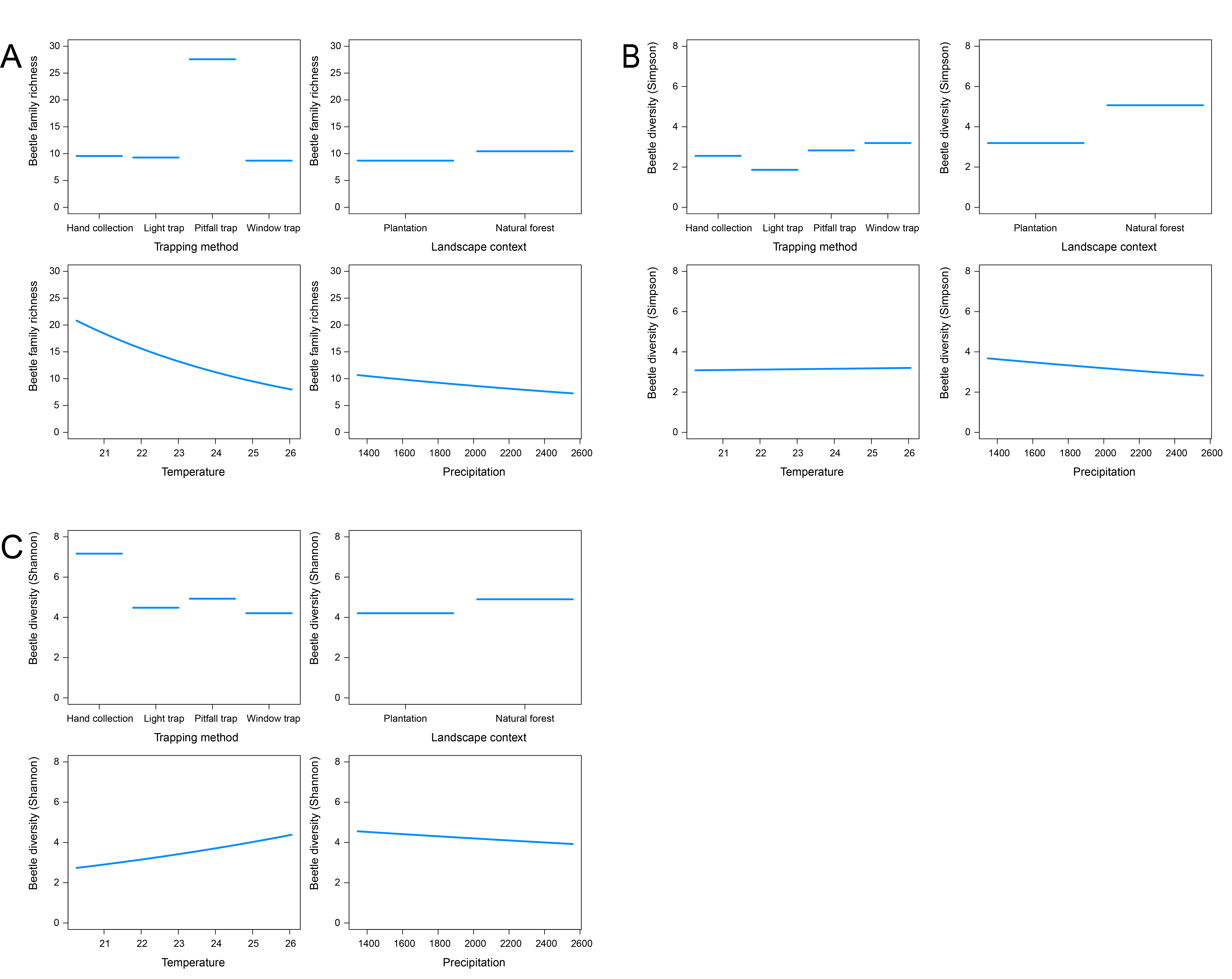


**FIGURE S2:** Partial dependence plots showing the predicted beetle richness (A), Simpson diversity (B) and Shannon diversity (C) for different levels of the four response variables tested. Only the effects of trapping method on richness and of landscape context on Simpson diversity are significant at the α=0.05 level.
